# “Putting your own oxygen mask on first”: a qualitative study of siblings of adults with anorexia or bulimia

**DOI:** 10.1186/s40337-021-00440-6

**Published:** 2021-07-08

**Authors:** Jannike Karlstad, Cathrine F. Moe, Mari Wattum, Ragni Adelsten Stokland, Berit S. Brinchmann

**Affiliations:** 1 Faculty of Nursing and Health Sciences, Nord University, Bodø, Norway; 2 Nordland Hospital Trust, Bodø, Norway; 3 KUN Centre for Equality and Diversity, Nordfold, Norway

**Keywords:** Anorexia nervosa, Bulimia nervosa, Siblings, Family relations, Qualitative research, Grounded theory

## Abstract

**Background:**

In families where one of the siblings has an eating disorder (ED), research indicates that the siblings without eating disorders (EDs) experience insufficient care and negative changes in family life. The illness then takes up a great deal of space within the family. Support from the siblings without EDs is considered to be important for the recovery of the sibling with ED. A key issue is how to involve siblings without EDs in treatment and establish what kind of support they themselves need. A majority of the research on EDs and family has focused on children and adolescents with EDs. The aim of this study is to expand knowledge about the experiences and coping strategies of sisters and brothers of adult women with anorexia nervosa or bulimia nervosa.

**Methods:**

This qualitative study used a constructivist grounded theory approach. Individual semi-structured interviews were conducted with 10 sisters and brothers (aged 20–31 years). They were recruited from eating disorders and general psychiatrics units and from user organisations for patients with eating disorders within Norway. An iterative process of data collection, coding and analysis was employed in order to generate a theory about these participants’ experiences and strategies.

**Results:**

The core category is “put your own oxygen mask on first”. It indicates that the siblings realize that they need to take care of themselves first, in order to be able to stay involved with their sister with the ED. The three subcategories; “taking a new role”, “distancing” and “adapted care” describe how the siblings handle their situation as the relatives of adult sisters with an ED. The siblings without ED became more responsible and independent and assumed a caregiving role. They downplayed their own needs to reduce their parents’ burden. This new role became difficult to fulfil and the siblings experienced that over time they needed more distance. Eventually, they developed a more manageable way of caring for their sister.

**Conclusions:**

The findings suggest that the ED challenged the boundaries within the family. The siblings without ED assumed a caregiver role, gradually leading to exhaustion and the need to distance from the sister with the ED, to take care of themselves.

## Plain English Summary

Siblings in families where one sibling has an eating disorder have reported experiencing a lack of care and negative changes in family life. They felt that the illness took up a great deal of space. This qualitative study explores the experiences of siblings of adult women with anorexia or bulimia. Ten individual interviews with brothers and sisters were conducted. The participants were recruited from eating-disorder and general psychiatrics units and from user organisations for eating disorders within Norway. The age range was 20–31 years. Findings from this study suggest that the character of the illness challenged the whole family. Participants reported that they became more responsible and independent. Some became caregivers for their ill sister and downplayed their own needs to reduce their parents’ burden. A majority of the siblings said that they experienced exhaustion after taking care of their sibling for a while and needed distance from their ill sister to take care of themselves. Eventually, they developed their own manageable ways of caring for their sister with the illness.

## Background

During recovery from an eating disorder (ED), support from siblings has been shown to have significant impact on the process [1]. Research on siblings of a family member with an ED has identified issues such as negative changes in family life, finding that the illness took up a great deal of space within their family and experiencing that they did not receive sufficient care themselves [2–4]. In 2020, a literature review on the siblings of individuals with EDs was carried out. It aimed to identify this population’s needs, in order to facilitate prevention and treatment. Siblings without EDs did not want to burden their parents or seek professional help. However, this group was considered vulnerable and mental health-professionals should be more proactive in identifying their needs and challenges [5].

Central coping strategies reported by siblings of individuals with an ED, were externalization of the illness from the sibling affected by ED and creating distance from the sibling with the illness [3, 4]. A much requested need among these siblings was information to better understand and manage the illness [3, 4]. Information and support in coping with the impact of the illness were considered important also for siblings of individuals with schizophrenia and psychosis. Caregivers’ tasks and responsibility for their sibling with the illness were often ongoing for years for the siblings of individuals with ED, schizophrenia and psychosis [3, 6, 7]. The relationship with the sibling with the illness often influenced their well-being as adults, even though sibling contact was reduced as the siblings without ED became adult and moved away from home [4, 6–8]. In general, siblings of individuals with severe psychiatric illnesses have been acknowledged as significant contributors to the care, and social and emotional support, of a brother or sister experiencing psychiatric illness. At the same time, these caring siblings have been largely invisible to the health services [9, 10].

A majority of the research on EDs and family has focused on children and adolescents with EDs [11–16]. Less attention has been paid to the families of patients with long-term EDs (commonly defined as illness with a duration of at least seven years [17]). The duration of the illness was found to have a significant impact on the experiences of family members [18]. Many of those suffering from an ED stayed at home with their primary family longer than their peers [19]. Families of relatives suffering from EDs of long duration were in need of appropriate professional support, to help them face the challenges posed by the ED over a long period of time [20]. The most difficult aspect for siblings of adults with long-term EDs was the siblings’ with the illness lack of motivation to recover. The siblings’ denial of the severity of the illness, accompanied by a tendency to try and rationalise it, caused feelings of helplessness. The siblings of adults with long-term EDs also reported positive aspects, such as increased understanding of psychiatric illness and strengthening of the sibling relationship [4].

A key message from a meta synthesis in 2017, on caring for or living with an individual with an ED, was that siblings were still often overlooked by researchers and clinicians [21]. References to siblings without EDs have mainly been about their contribution to their siblings’ with the illness recovery [5]. A greater focus on the siblings of individuals with EDs of long duration is required [4, 18, 20]. The aim of the current study is to expand knowledge about the experiences and coping strategies of sisters and brothers of adult women with anorexia nervosa (AN) or bulimia nervosa (BN). What are their experiences and what strategies do they use to handle their situation as siblings of adult sisters with AN or BN? The study focuses on participants’ daily life experiences.

## Methods

### Design

A constructivist grounded theory approach [22] was chosen to further explore the experiences and strategies of siblings of adult women with an ED. The focus is on women, as they are reported to be the most frequent patients [23]. Studying a mixed group of siblings of men and women could be problematic, as the two groups may perceive eating disorders differently. The constructivist version of grounded theory differs from the original version, which emphasized that the researcher should have an objective approach to the data [24, 25]. Grounded theory is appropriate when the goal is to generate a theory that explains a phenomenon where knowledge is limited [26], as in the situation where the object of study are the siblings of individuals with an ED [4, 21]. To generate a theory about these participants’ experiences and strategies, an iterative process of data collection, coding and analysis was employed [24, 26].

Two co-researchers (MW, RAS) were involved in the project as consultants. One has personal experience of suffering from an ED; the other is the mother of an individual with an ED. They were involved in developing the research questions, writing the interview guide and in the final process of analysis.

The constructivist approach includes the researcher’s preconceptions and viewpoint. Reflexivity is required throughout the research process by explicating taken-for-granted assumptions, while being aware of how hidden beliefs can enter this process [27]. As the first author has clinical experience with patients with EDs, this approach was considered essential. Co-researchers’ insights enabled the author to develop and expand upon her own original thoughts and perceptions, which were based on analysis of the data. In constructivist grounded theory it is acknowledged that what one discovers in the data is part of one’s own perspective, but that one should not see this as the one true explanation of the data [22].

### Participants

Participants were the brothers and sisters of adult women (over the age of 18 years) with AN or BN. For the majority of the siblings affected by ED, onset of the illness took place in their early teens. A few were in their early twenties. Long duration (± 10 years) of illness was common for the majority of the group, while a minority had been ill for a shorter term. All had at some point received treatment at inpatient or outpatient clinics and some were still being treated. None of the siblings were referred to as fully recovered from the ED. Most fluctuated between better and worse periods. All the siblings but one no longer lived with their sister with ED. Participants were recruited from one inpatient and three outpatient eating disorders and general psychiatrics units and two organisations providing information and support to patients and families about EDs. The units conveyed information about the project to prospective participants, who signed up voluntarily. Participants were recruited from different counties within Norway. Ten individual interviews were conducted, with three brothers and seven sisters, from eight families. Table 1 describes characteristics of the participants.

**Table 1:** Characteristics of participants

Participant pseudonym	Age of participant	Age of sister	Diagnosis of sister	Number of siblings
Ada	31	30	BN	4
Anne	21			
Adam	26			
Beatrice	30	24	AN	2
Cecilie	29	24	AN	2
Dina	20	20	AN	2
Eva	22	27	AN	3
Fred	25	20	AN	2
Gabriel	25	24	BN	3
Helene	22	25	AN	3

### Procedures

#### Data collection

Semi-structured interviews [28] were conducted using an interview guide with a few predetermined open questions. Interviews were started by asking participants to describe their experiences of being the sister or brother of an adult sister with an ED. The participants were encouraged to talk about what felt important to them in this context. They were asked about what effect living or having lived close to a family member with this illness had on themselves and the family. During the interviews, participants were also asked about coping strategies for handling the situation as the sibling of a sister with an ED and their experience of the health services in this context. The guide was slightly adjusted as new themes arose [22, 28]. When a new theme emerged during the first interview, for example “taking on a new role”, the theme “role” was then incorporated into the interview guide.

All the interviews were audio recorded and transcribed verbatim. Each lasted between 28 and 55 min. The participants’ quotes used in this article have been translated from Norwegian. Six interviews were conducted face-to-face. The remainder were conducted by telephone due to Covid-19 travel restrictions. The participants preferred telephone interviews to the option of video conferencing interviews.

#### Data analysis

The data were analysed following the principles of constructivist grounded theory [22]. First, the transcriptions were initial coded, sticking closely to the data by studying words, lines and segments. In vivo codes, i.e. participants’ own terms, were used where possible. To synthesize and conceptualize larger segments in the data, the process of focused coding was begun and a core category constructed. After the first interviews, reflections on codes appearing in data and ideas for an initial analysis were written down in memos. For example, one memo written after the first interviews was about “distancing”. What did “distancing” mean in this context? Was it a defence mechanism? The focused code “distancing” emerged from initial codes such as “pulling out”, “pushed it away”, “did not want to push her” and “concentrated on myself”. After further interviews the categories eventually became thematically saturated, as new data did not add new characteristics to the categories. The rich data from the interviews saturated the characteristics within the categories [29]. To ensure a consistent process, all the authors participated in the final analysis, by meeting and discussing the results.

Seven of the participants agreed to read a summary of the preliminary analysis and respond to it. The aim was to see whether they could recognize themselves in the material or felt something had been left out. The responses from the participants were taken into consideration in the final analysis.

#### Ethics

This project was approved by the Regional Committees for Medical and Health Research Ethics, REK Vest, Norway (2019/762), and by the Norwegian Centre for Research Data, Norway (2020/222970). Participants gave their informed, written consent to voluntary participation in the project and had the opportunity to withdraw at any time. As requested by Ethics Committee prior consent was required from the sibling affected by the eating disorder. Participants’ confidentiality was protected by anonymising the data and information about their identity was stored securely, in accordance with data legislation and university procedures. The participants’ names in the results section are pseudonyms.

## Results

*An incomprehensible illness that challenged the boundaries within the family* was identified as the siblings’ main concern*.* All the siblings found the ED difficult to understand and felt that the severity and character of the illness affected the whole family and challenged its boundaries. This sister said: *“*… *suddenly it was okay to shout and scream at home, over every little thing. It is as though this one person (the sister with the illness) changed the rest of the family*… *It does not feel right, at least not when it is so difficult to understand” (Cecilie).* How the siblings experienced and adapted to the ED varied according to the severity and duration of the illness and how involved they had been, or still were, with the sibling with the illness. All the participants started by talking about how the illness began. In most cases the illness arose when their sisters were in their teens or early twenties. Most of the siblings described a long-lasting situation that was still ongoing. A majority had lived together with the sister while she was ill, though only one was living together with the sister at the time of the interviews.

### “Put your own oxygen mask on first”

To manage the main concern, the core category “*put your own oxygen mask on first”* was identified. This metaphor expresses the idea that before you can help others, you first need to take care of yourself. A majority of the participants said that they had provided more support and help in the early phases of their sisters’ illness, than in the later stages. Most of them had experienced that over time they might have made too much effort in supporting their ill sister, and that they had to take care of themselves as well: *“I had to try to help myself too. I wanted to take the load off my mom by being there for my sister, but it became too hard and I needed to get away” (Ada).* All the participants who responded to the preliminary findings thought that the metaphor “putting your own oxygen mask on first” could appropriately describe their situation. The three subcategories: *taking a new role, distancing* and *adapted care* explain how the siblings handled this situation.

### Taking a new role

The siblings experienced that the ED took up a great deal of space in the family, as the ill sister required everybody’s attention. As a result, the siblings without EDs often assumed a new role in the family. They started to take on practical duties at home and accepted additional responsibilities. For some this was more than they wanted, although they wished to spare their parents and take some of their burden, as well as supporting their sibling with the illness. *“I performed the role of adult to a great extent.* … *dad was upset, my sister was upset, and I was the one comforting them” (Helene).*

The whole family’s focus on the sister with the illness, especially by the parents, made the other siblings become more independent. Several of the participants told that during the first years of their sister’s illness there was little room for the siblings to have their own adolescent lives. The feeling of being in the way and having to accommodate their lives to the sister with the illness, followed several of them into adulthood. Nor did they want to cause their parents additional worry: *“I do not feel as though I can ever be sick, as it would become an additional burden on my mom and dad” (Anne).* The siblings seemed to find themselves more in the role of caregivers than siblings: *“You could say that one becomes very protective and motherly” (Eva).* The siblings’ statements indicated that they had taken on considerable parental responsibilities. Some of the siblings described ending up in the role of mediator between their sister and their parents. This sister was expected to report to the parents on the behaviour of her sister: “*I became responsible for making sure she ate enough.* … *to monitor and control what she ate. After a while, I think my parents realized that it was too much for me to manage all of the time” (Dina).*

The sibling described that the relationship had started to change character as time passed and the changing roles evolved. Some of the sisters especially experienced that they grew apart and some of them felt that they “lost” a close friend in the ill sibling. In their responses to the preliminary analyses some wrote that they had learned to see their situation as a process.

### Distancing

Most of the siblings experienced that over time they had needed to distance themselves from their sibling with the illness. In a process that gradually developed over time, theses siblings recognised that they had become too involved and had put too much effort into supporting her. Some felt aggression and frustration toward their sister with the illness. The siblings had started to feel exhausted and realized they had to take care of themselves too. Some of them moved away, while others reduced contact in other ways. A few of the siblings still wanted to stay close to their sister with the illness, as close friends. At the same time, they wanted to distance themselves from their roles as quasi-professional carers.

Most of the siblings needed distance in order to take care of themselves: *“She has been ill for so many years that you feel you have to distance yourself a little, or you are going to go crazy yourself” (Helene).* Distancing could be seen as a selfish act: *“There is not much caring in that, other than maybe for myself” (Beatrice).* Several of the siblings expressed feelings of guilt about the distancing and less contact meant that they worried about not knowing how their sisters were doing. One sister found that she needed distance to be able to continue to care for her sister: *“My experience is that I thought I could give her all I had. Perhaps I should have found a middle way between comforting her and being there all the time, before pulling away completely. Better to keep some distance and be able to stay the course” (Ada).*

Uncertainty as to how to relate to the illness could also lead to distancing, through being afraid to say or do something wrong, as with this brother: *“I learned to keep some distance when she did not have a very good day” (Gabriel).* For some, it had been difficult to gain an understanding of the eating disorder. This then made it difficult to know how to support the sibling with the illness. All the siblings emphasized the need for general information about the illness from the health services or in school, but only a few recalled having received such information. Several of the siblings were careful in their choice of words, in order to maintain a calm atmosphere. They kept a certain distance by not saying what they really felt. In terms of endeavouring to do the right things, their involvement with the sister with the illness could be a balancing act: *“Keeping the head cold and the heart warm” (Beatrice),* as this sister described it*.* Some said they had wanted to be more involved in the treatment. Only a few of the siblings said that they had received any kind of support from the health services to help them cope with their own demanding situation. Several of the siblings requested sibling-focused support groups as one possible intervention.

### Adapted care

The siblings described that they had adapted their care for the sibling with the illness, depending on the severity and duration of the illness and any relapses during recovery. Often, the nature of the care changed and the relationship could appear more superficial. Some maintained most of the contact by telephone, others met in particular settings and engaged in certain recreational activities together. The siblings described the relationship with their sisters as a cyclical process over time: from being close to more distant and from being protective to requiring the sister to take more responsibility for herself.

Talking about the illness and sharing deeper feelings became less frequent. *“Now our relationship is more like before, we talk about sports and things like that.* … *I find it difficult when we talk about her problems. We have done it, but I do not bring it up every time we talk” (Ada).* Some of the siblings talked about periods when they worried more about their sisters, such as when relapses or hospitalisations occurred. For this sister, the talk about illness was downplayed out of consideration for her sister: *“Maybe, in certain periods when I see that she is doing worse, those worries appear. But I do not want to bother her with my concerns” (Beatrice).*

Several of the siblings had found their own ways of showing that they cared—ways that seemed manageable to them. The siblings did many situation-specific things to show that they cared. This brother tried to help his sister around mealtimes: *“You knew what happened when she left the table, so then I tried to plan my visits to the toilet. I would go there just to let her keep the food in her stomach a little longer” (Adam).* A couple of the siblings helped their sister by paying for petrol, so that she did not waste energy on walking or would not freeze while waiting for the bus. Another brother tried to contribute to his sister’s safety: *“She reaches out for me when she needs someone safe. … So sometimes when she has had an anxiety attack, she calls me to help boost her confidence” (Gabriel).*

Two of the siblings had found that the time had come to set some boundaries for their sisters with the illness: *“That you do not just surrender everything for her” (Gabriel)* and *“You are supposed to understand that they are ill, sure, but they do not have to behave just as they like” (Cecilie).* One way of showing that they cared was to require the sister to take some responsibility. A couple of the siblings became parents. This led to the attention that previously revolved around the sister’s illness, being refocused on the sister’s new role as aunt: *“So a lot of the communication between us was suddenly about her becoming an aunt. And through that I found a way to show her that I care” (Beatrice).*

One of the sisters that responded to the preliminary analysis had experienced that when the process of managing the illness took over the whole family, she learned to hide her own feelings. She said that she has continued to pretend, in order to keep peace within the family.

## Discussion

Our findings suggest that the boundaries of the participants within their families and their roles shifted. The participants identified themselves in a caregiver role to a greater extent than they considered normal for siblings or children, reflected in the responsibilities and duties they took on in the family. Within a traditional family system, one subsystem comprises the parents, one comprises the children and another the siblings. Boundaries are invisible barriers which regulate the contact between subsystems within the family and help to maintain a certain hierarchy between the subsystems [30]. Hierarchically, the siblings placed themselves at the same level as the parents, while they perceived that the siblings with the illness needed the attention and support like a child. Having greater responsibility and being undemanding are reflected in previous research on siblings in the same situation [2, 4]. These siblings found themselves in multiple roles or torn between roles [2], as in the current study where the boundaries appeared indistinct to the siblings and they experienced role confusion. Absence of firm boundaries within a family could also entail positive components and family members could provide unconditional care without being hindered by boundaries [30]. However, the downside described by the siblings in the current study was that to varying degrees they lost the capacity to take care of themselves.

Due to the complexity, severity and duration of the illness a majority of the siblings had experienced exhaustion after having performed in their new roles for a while. They started distancing themselves from the sister with the illness and the new family system that had evolved. Previous research has revealed a tendency, when being overwhelmed, to try and separate the illness from the person by establishing distance [3–5]. A few of the participants in the current study said they did not want to create distance. This might be due to the nature of the relationship, or because their sister’s illness was of lesser duration than the other ill siblings in the study. A wish to spend as much time as possible with the sibling with the illness was also identified in a few participants in a study on youth siblings [3]. Family distancing has often been seen as synonymous with relationship dissolution, while others have argued that family distancing can be more multifaceted. Rather than distancing themselves, family members often stay in relationships which can lead to negative outcomes for the relationship and those involved. Distancing may be essential for family functioning, and is not necessarily seen as a problem [31]. In the current study the participants explained their need to create distance not only for self-protection, but also to help them to sustain their involvement with their sibling with the illness. Previous research on adult siblings has found that important reasons for not managing to stay emotionally involved included the siblings’ with the illness lack of motivation to recover, certain eating behaviours and their rationalizations of the illness. The siblings learned how to create a firm boundary between themselves and their sibling with the illness for the purpose of self-preservation [4].

Processes of family distancing can be seen as a healthy solution to an unhealthy relationship. Distancing could be positive for one family member, but problematic for others, hence feelings of ambivalence could be present [31]. Many of the siblings expressed feelings of guilt for not being there enough, while at the same time needing a break. Previous research also found that the siblings’ efforts to protect themselves resulted in feelings of guilt [1, 4].

Distancing can be done in several ways; moving away, reducing the amount of contact, or ignoring expectations [31] and staying busy with one’s own life [4]. Creating distance can also be achieved by avoiding personal matters in conversations [30]. By distancing in several ways, the siblings explained that they found more manageable ways of caring.

Hopes of recovery appeared to be an important strategy to help siblings to overcome their eating disorders [3–5, 32]. For the participants in the current study, this strategy was not perceived to be applicable, as the illnesses were mainly of long duration and hopes of recovery were weakened by recurring relapses. Previous research reports suggested that ability to cope is facilitated by a better understanding of the illness, which health professionals are then expected to provide [5]. Participants also expressed a need for professional counselling for themselves [2].

The siblings of individuals with severe psychiatric illness are in general reported to be in need of information and support for two reasons. The first is to maintain their own wellbeing; the second is to help them support their ill sibling and family [10]. Several of the siblings in the current study requested sibling-focused support groups and this has also been reported in previous research [4, 32]. One brother said that he could have made good use of “a little manual” explaining how to handle this situation.

### Strengths of the study

A strength of this study was its inductive and open approach, which encouraged the participants to emphasize what is important to them in this context [22, 28]. By reporting the experiences of adult siblings acting as caring relatives in the ED situation, this group’s support needs are highlighted—information that is highly relevant for the health services. The co-researchers ability to view the results from a different angle, based on their own experiences, was of great value during the project. Their involvement throughout the research process strengthened the credibility of this study [33, 34].

### Limitations of the study

When recruitment is based on self-selection, it is possible that the individuals who volunteer share certain characteristics, or have some particular reason for wanting to participate. In this study, as in many others that examine the everyday burdens and health effects of caring for individuals with severe psychiatric illnesses, the majority of the participants were women [21, 35]. On the basis of the limited sample of three brothers and seven sisters, no considerably differences between the two groups’ experiences as siblings of an individual with ED could be reported. As some of the interviews were conducted by telephone, the communication may have differed from those held face-to-face, because it is not possible to read body language over the telephone.

### Future research

Research should continue to explore the situation of siblings as the relatives of individuals with psychiatric illnesses, especially adult siblings as it is they who have received the least attention [4, 8]. Long duration of the illness and the relatively scant hope of recovery appear to be challenges particularly for adult siblings. The siblings of individuals with EDs share similar experiences and needs with siblings of individuals suffering from other psychiatric illnesses [6, 9, 10]. However, the current study shows that dealing with the complex character of the eating disorder can bring some specific challenges. The siblings’ experiences of challenges regarding boundaries in the family and they assuming a caregiver role, and the ambivalence about being torn between the need for self-care and care for the sibling with the ED, are specific elements that need further exploration.

### Implications

There needs to be more focus on the siblings without EDs, and more consistent support for them, while the ill sibling is being treated [32]. The siblings of adults with EDs need better information and counselling about the illness, as well as better personal support when they face this situation. The siblings’ experiences with their sibling with the illness, including their advice on treatment, should be listened to by the health services. Adult siblings take on great responsibility for their siblings with EDs, often for long periods of time.

## Conclusions

This study aimed to explore the experiences and strategies of sisters and brothers of adult women with AN or BN. Findings suggest that the siblings often assumed great responsibility when caring for the ill sister. After a while, the siblings experienced exhaustion. They then needed to create distance from the ill sister, in order to take care of themselves. Eventually they found their own more manageable ways of caring for their ill sister. The results emphasise that the siblings of an adult with ED need support, information and counselling about the illness from the health services.

## Data Availability

The dataset collected and analysed in the current study are not publicly available due to ethical restrictions to maintain the participants’ anonymity. The corresponding author can be contacted on reasonable requests regarding the dataset.

## Abbreviations

AN: Anorexia nervosa
BN: Bulimia nervosa
ED: Eating disorder
